# Anemia in Crohn’s Disease—The Unseen Face of Inflammatory Bowel Disease

**DOI:** 10.3390/medicina57101046

**Published:** 2021-09-30

**Authors:** Mihaela Dranga, Lucian Vasile Boiculese, Iolanda Valentina Popa, Mariana Floria, Oana Irina Gavril, Oana-Bogdana Bărboi, Anca Trifan, Cristina Cijevschi Prelipcean, Cătălina Mihai, Otilia Gavrilescu

**Affiliations:** 1 Medicale I Department “Grigore T. Popa”, University of Medicine and Pharmacy, 700111 Iasi, Romania; mihaela_dra@yahoo.com (M.D.); floria_mariana@yahoo.com (M.F.); ir.ungureanu@yahoo.com (O.I.G.); oany_leo@yahoo.com (O.-B.B.); ancatrifan@yahoo.com (A.T.); otilianedelciuc@yahoo.com (O.G.); 2 Institute of Gastroenterology and Hepatology, St. Spiridon Emergency Hospital, 700111 Iasi, Romania; cristinacijevschi@yahoo.com; 3 Department of Medical Informatics, Biostatistics, Computer Science, Mathematics and Modelling Simulation “Grigore T. Popa”, University of Medicine and Pharmacy, 700111 Iasi, Romania; lboiculese@gmail.com; 4 Medicale II Department “Grigore T. Popa”, University of Medicine and Pharmacy, 700111 Iasi, Romania; iolivp@gmail.com; 5 Department of Internal Medicine, Dr. Iacob Czihac Emergency Military Hospital, 700483 Iasi, Romania; 6 Department of Internal Medicine, CF Hospital, 700506 Iasi, Romania

**Keywords:** Crohn’s disease, anemia, quality of life

## Abstract

*Background and Objectives*: Anemia is the most frequent complication of inflammatory bowel diseases. Clinically, anemia can affect important quality-of-life (QoL) components, such as exercise capacity, cognitive function, and the ability to carry out social activities. The disease activity has a significant impact on QoL, mainly due to clinical manifestations, which are more severe during the periods of disease activity. Our aim was to estimate the impact of anemia on QoL in patients with Crohn’s disease. *Material and Methods*. We made a prospective study on 134 patients with Crohn’s disease (CD) in a Romanian tertiary center. The CD diagnosis was established by colonoscopy and histopathological examination. In particular cases, additional examinations were required (small bowel capsule endoscopy, computed tomography enterography, and magnetic resonance enterography). Anemia was defined according to the World Health Organization’s definition, the activity of the disease was assessed by Crohn’s disease activity index (CDAI) score, and the QoL was evaluated by Inflammatory Bowel Disease Questionnaire 32 (IBDQ 32). *Results*: 44.8% patient had anemia, statistically related to the activity of the disease and corticoids use. We found a strong association between QoL and disease activity on all four sub-scores: patients with more severe activity had a significantly lower IBDQ (260.38 ± 116.96 vs. 163.85 ± 87.20, *p* = 0.001) and the presence of anemia (127.03 vs. 148.38, *p* = 0.001). In multiple regression analyses, both disease activity and anemia had an impact on the QoL. *Conclusions*: Anemia has high prevalence in the CD in northeastern region of Romania. Anemia was more common in female patients, in patients undergoing corticosteroid treatment, and in those with active disease. Both anemia and disease activity had a strong negative and independent impact on QoL.

## 1. Introduction

Anemia is the most frequent complication of inflammatory bowel diseases (IBDs), with a prevalence that varies between 6% and 74% [1]. Anemia in IBD is a complex process, engaging multiple factors that contribute to its emergence. The most frequent cause is determined by the iron deficit (chronic loss, poor absorption due to mucosa inflammation and ulceration, and short bowel syndrome); however, there may be other etiological factors, such as vitamin B12 and folic acid deficiency, the effects of proinflammatory cytokines, or hemolysis and certain drugs [2].

Health-related quality of life (QoL) was defined as the functional effect of a disease and its treatment on a patient, as perceived by the patient. The patient’s perception on his/her own health is vital for the understanding of the way in which (s)he carries out activities at home, at school, or at work [3]. IBD symptoms (abdominal pain, diarrheal stools or constipation, nausea, weight loss, and marked asthenia) are manifested especially in disease activity, with the activity and severity of the disease being the main determinants of QoL [4,5,6].

The need for a QoL assessment emerged in order to reach the medical goal of improving the general well-being of patients. This is useful in quantifying the effects of the treatment, describing the nature and severity of a condition and assessing prognosis, but also in identifying the most appropriate type of therapy, comparing the benefits and side effects of different types of treatment [7]. QoL assessment tools have been increasingly used over the last period. They complement the clinical and biological evaluation of various disorders, the assessment of the quality of the medical services, the medical care needs, the effectiveness of diagnosis or therapeutic interventions, and the estimation of financial costs [8].

Clinically, anemia can affect important QoL components, such as the exercise capacity, the cognitive function, and the ability to carry out social activities [9,10]. In addition, anemia increases the number of hospitalizations and even mortality rates [11,12,13].

Starting from these prerequisites, we conducted a study to estimate the impact of anemia on QoL in patients with Crohn’s disease (CD).

## 2. Materials and Methods

A prospective study was conducted over a period of 3 years (1 January 2016–31 December 2019). The study included 134 patients with CD—both patients with a history of CD and patients who were at their first diagnosis—who were evaluated during this period in a Romanian tertiary center.

At the time of the assessment, the following parameters were recorded: age, marital status, occupational status, and smoking obtained from anamnesis. A complete clinical examination was performed for each patient, investigating, in particular, abdominal sensitivity, the possible presence of the palpable masses, and articular and mucocutaneous modifications, which could point to an extra-intestinal manifestation. Upon hospitalization, hematological and biochemical tests were performed, investigating specifically hemoglobin and inflammatory markers. The positive diagnosis and the extent of the disease were established by colonoscopic examination and confirmed by the histopathological examination. All patients underwent total colonoscopy, and in the cases where the small intestine involvement was suspected, additional examinations were required (small bowel capsule endoscopy, computed tomography, and magnetic resonance imaging). The extension and behavior of the disease were determined according to the Montreal classification. The clinical activity was evaluated according to the Crohn’s disease activity index (CDAI). Clinical remission was defined for a CDAI score of ≤150.

Patients associated with hematological disorders (anemia), kidney and liver disorders, and neoplasm, which might have influenced the studied parameters, were excluded from the study.

Anemia was defined according to the World Health Organization at a concentration of Hb < 12 g/dL in non-pregnant women and <13 g/dL in men [13].

QoL was assessed by means of the IBDQ-32 questionnaire (Inflammatory Bowel Disease Questionnaire). This is a well-validated disease-specific instrument to measure QoL in patients with IBD and has previously been translated and validated for its use in Romanian patients [14]. The questionnaire has 32 questions structured into four fields: IBDQ1—gastrointestinal symptoms (diarrheal stools, abdominal pain, rectorrhagia, and rectal tenesmus), IBDQ2—systemic symptoms (fatigue and sleep disorders), IBDQ3—emotional functions (depression, irritability, anger, and sexual activity) and IBDQ4—social functions (absenteeism and affected social status). The responses in the questionnaire were graded from 1 (the worst) to 7 (the best). The total score is included in the range 32–224. Moreover, we computed the sub-scores for each distinct field (emotional functions, 12–84; gastrointestinal symptoms, 10–70; systemic symptoms, 5–35; and social functions, 5–35). The lower this score is, the more QoL is affected.

### 2.1. Statistical Analysis

Statistical application SPSS 18.0 for Windows was used in order to process the data. We first described the variables by means of average, standard deviation, minimum and maximum (in one table also the median) for continuous data, and absolute frequency and percentage for categorical ones.

The hypothesis tests, used for statistical comparisons, turned to account the ANOVA method, the Student’s *t*-test where normal approximation is acceptable, as well as the Mann–Whitney U test and Kruskal–Wallis for score variables. For categorical data, the Chi-square test was applied.

The standard significance cutoff was 0.05, and it was needed to decide on alternative hypothesis conclusion.

### 2.2. Ethical Aspects

All patients enrolled in the study signed an informed consent, which explained details about the purpose of the study, its methodology, and the risks and benefits involved in the study, as well as information regarding the confidentiality of the results. The study was conducted according to the Declaration of Helsinki and approved by the Ethic Comittee of “Grigore T. Popa” University of Medicine and Pharmacy in Iasi (code 116, 10 December 2015).

## 3. Results

The study included 134 patients with CD. The average age of the patients was 43.57 ± 15.57, and the patients were equally male and female. Most patients came from the urban environment. A quarter of the patients were in remission. The most frequent disease form was colonic and ileo-colonic CD and inflammatory phenotype (Table 1).

Anemia was more frequent in women and in the patients who underwent corticosteroid therapy. There were statistically significant correlations between the presence of anemia and the CDAI value. Moreover, anemia was significantly more present in patients with active disease (Table 2).

QOL was significantly influenced in the patients presenting anemia and those with active disease (Table 3).

In the patients with anemia, QoL was affected both globally and at the level of the subfields, assessed by means of the scores obtained in the IBDQ-32 questionnaire (Table 4).

When analyzing the univariate influence that the two factors (disease activity and anemia) had on the QoL, we noticed that patients with anemia who were in remission had IBDQ3 and IBDQ4 significantly affected compared to patients without anemia (Table 5).

Finally, in order to check the codependence of the variables activity and anemia in terms of QoL impact, we used multiple regression. The effect of the disease activity (CDAI) on QoL is important in multiple regression. The reduction of the coefficient for the adjusted form is not significant—for example, for total IBDQ from 0.132 to −0.112, thus, with (0.132–0.112)/0.132 ×100% = 15.1%. Anemia had a lower quantifiable effect on QoL, quantified by total IBDQ (Table 6).

## 4. Discussion

The data regarding the prevalence of CD in the world are heterogeneous, and they differ depending on the studied region and the studied population (hospitalized or ambulatory patients). The first published data estimated an anemia prevalence of 74% of the studied population [15]. A recent meta-analysis estimates the prevalence of anemia to a 27% ratio in patients with CD [15]. In hospitalized patients, anemia prevalence reaches 70%, while, in ambulatory patients, this prevalence drops to 15% [16]. Anemia prevalence was 7% in Sweden, 9% in the Netherlands, and 40% in the United Kingdom [16]. According to recent data published for the western and central regions in Romania, anemia prevalence in CD patients is as high as 35.65% [17]. In the northeastern part of Romania, where the present study was conducted, the data are relatively old (2012). These data estimated an anemia prevalence of 46.15% [18]. In our study, anemia prevalence was 44.8% higher than in other regions of the country. This could be explained by several factors: this is a poor region; the study was conducted in a tertiary establishment in which severe forms of the disease are treated; and the study was conducted on hospitalized patients, and three-quarters of them had flare-ups. Nevertheless, we can notice a decrease in prevalence over the last years, due to a more efficient management of this manifestation.

Specialized studies demonstrated that female gender is associated with an increased risk of anemia occurrence [19,20]. However, this view is not unanimously accepted; there are studies in which no statistical differences were found between the two genders regarding the occurrence of anemia [21]. In our study, a significantly higher incidence of anemia was noticed in women as compared to men. The prevalence of increased anemia in women can be explained by the presence of other losses, such as menstruation.

The study did not aim to analyze the influence of treatment on the rate of anemia, and, consequently, no data were collected on treatment doses, medication administration route, treatment duration, or treatment administration in remission. However, we noticed a significantly higher prevalence of anemia in patients who were given corticosteroid treatment. This could be explained by the fact that most patients were in activity, and corticosteroid therapy was needed to induce remission. Under these circumstances, we consider that anemia is secondary to disease activity (at the level of the mucous membrane or inflammation) and not due to medication itself. Moreover, studies in the field have not shown any correlations between medication and anemia [16].

Similar to other studies in the literature, our study also demonstrates a higher rate of anemia in patients with active disease. Decreased iron level, which, in time, may lead to iron deficiency due to ineffective erythropoiesis, is caused by decreased intake (inappetence and digestive intolerance) [22], chronic intestinal loss [23], and, rarely, by decreasing iron uptake (lesions at duodenal or jejunal level). Moreover, during activity flares, inflammatory anemia can occur, due to the modifications of cytokines and acute phase proteins. Besides the modulation of the iron homeostasis, the cytokines directly influence hematopoiesis by inhibiting the erythroid cells. Moreover, TNF α decreases iron incorporation into erythrocytes, thus leading to anemia [24]. It is noticed that patients with anemia had more severe forms of the disease (CDAI), with all of these mechanisms being more pronounced in these forms.

Most researchers suggest that the female gender tends to pay more attention to the disorders they suffer from, generally overestimating their symptoms, and being thus more affected by psychosocial factors [25,26]. Even in the studies assessing the QoL related to health in the general population, the female gender showed lower scores than the male gender [26,27]. Although more studies have shown that IBD has a higher negative influence on QoL in women [28,29,30], there were no statistically significant differences in total IBDQ-32 scores between males and females.

There are few studies on the QoL correlation with IBD location. The study carried out by Won Han S et al. demonstrated that QoL in IBD was mostly influenced by clinical manifestations and not by IBD localization [31]. These data are congruent with the results obtained in our study. The lesions localization and the disease phenotype do not significantly affect the QoL in these patients.

Similar to the literature data [32,33,34], the disease activity had a significant QoL impact estimated by IBDQ in the patients included in the sample. This is mainly due to clinical manifestations, which are more severe during the periods of disease activity. Additionally, disease activity disruptions may influence the onset or worsening of psychiatric disorders such as anxiety or depression, which is an additional factor impairing the QoL in these patients [35].

The increased frequency of symptoms is directly related to the impaired QoL, which is reflected in both in QoL assessment tests and in IBD specific tests.

Moreover, activity flare-ups seem to be closely related to the degree of fatigue and sleep disorders, the latter being independent factors influencing the QoL [36,37].

In our study, QoL was affected both globally (IBDQ) and in each of the sub-scores. The patients in activity flare presented more frequently gastrointestinal symptoms (diarrheal stools, abdominal pain, rectorrhagia, and rectal tenesmus) (IBDQ1), and had significantly increased fatigue and sleep disorders (IBDQ2). In addition, the emotional function was more frequently and more intensely affected (depression, irritability, anger, sexual activity) (IBDQ3), all of which lead to increased periods of absenteeism and, implicitly, social impairment (IBDQ4).

Another factor that significantly affected QoL in these patients was the presence of anemia. There are several studies in the literature that have shown that anemia has a negative impact on the QoL in patients with IBD. A study conducted in the US showed that, together with disease severity, osteo-articular extraintestinal manifestations, cardiovascular diseases, and age, anemia represents an important factor with a negative impact on the physical component of the QoL [38].

Another study assessed the influence of serum hemoglobin modifications on QoL scores in these patients and its correlation with IBD activity. The results of the study demonstrated that serum hemoglobin optimization improved QoL scores in IBD patients, independent of the disease activity [11].

Furthermore, Gisbert JP et al. showed that iron substitution treatment used for anemia correction in patients with IBD was associated with significant QoL improvement in these patients [12].

When analyzing patients by categories of activity, we noticed that patients in remission with anemia presented significantly more frequent and more important emotional symptoms (*p* = 0.008) compared to patients without anemia. Similarly, social life was more impaired in these patients (more frequent periods of absenteeism and social activities impairment) (*p* = 0.029) compared to those without anemia. This shows that anemia does not depend on the activity of the disease and its impact on QoL.

Later on, we performed a multiple regression data adjustment to reveal the effect that each of the two variables (disease activity and anemia) had on QoL. The effect of CDAI is also significant in univariate analysis and in the adjusted form (multiple regression). We notice that the effect of anemia is not always significant, but it has a measurable impact on total IBDQ. This is lower than the effect of disease activity quantified by the CDAI score, with the effects being independent from one another.

**Figure 1:**
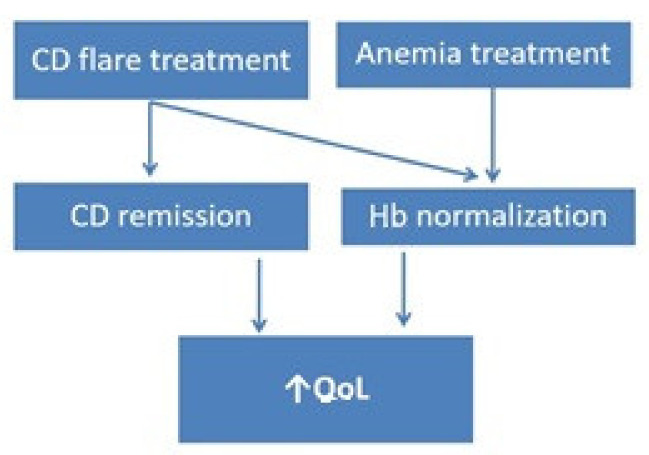
Practical management for improving quality of life in CD patients with anemia. CD: Crohn’s disease; Hb: hemoglobin; Qol: quality of life.

From a practical point of view, our study has demonstrated that both the treatment of flare and the treatment of anemia are needed to increase the QoL of CD patients (Figure 1).

### Strengths and Limitations

The study brings new data on the prevalence of anemia in the northeastern region of Romania, where the published data were rather scarce and old. The concept of QoL has been increasingly debated in recent years, being used in medicine and other fields. Improving QoL was and has remained an end objective in any medical activity. The study evaluates the impact that anemia and disease activity have on QoL in patients with CD. Compared to other studies in the literature, this research analyzes the codependence of the two factors in terms of QoL impairment by disease categories.

One of the limitations of the study consists of the fact that, being a transversal observation study, we did not assess the effect of the treatment by taking into account the treatment duration, dosages, and route of administration. Additionally, neither did we assess the effectiveness of the substitution treatment of anemia on the QoL, as this aspect exceeded the scope of our study. Another limitation of the study was the fact that we did not assess the endoscopic activity of the disease; given the fact that colonoscopic examinations were performed by a large number of investigators, the description protocols were not unitary, and many cases had small bowel involvement. Moreover, it is known that, in CD, there is a weak correlation between symptoms and endoscopic activity, which suggests that the latter has a low impact on QoL.

## 5. Conclusions

To conclude, anemia has high prevalence in the northeastern region of Romania. Anemia was more common in patients undergoing corticosteroid treatment and in those with active disease. Both anemia and disease activity had a strong negative and independent impact on QoL. In the case of patients in remission, emotional symptoms were more frequent and more prevalent in patients with anemia compared to those without anemia. Social activities were significantly modified in these patients; similarly, absenteeism periods were more frequent compared to the patients without anemia. Based on these data, in order to improve quality of life, we consider that anemia should be corrected according to the existing guidelines (ECCO) in all patients, not only in those with disease activity.

## Figures and Tables

**Table 1:** Clinical and demographic characteristics of participants included in study.

Characteristic	Computed from Whole Sample
Number of patients	134
Gender, *n* (%)	
Female	68 (50,67)
Age, mean ± SD (min–max)	43.57 ± 15.57 (19–82)
Marital status, *n* (%)	
Married	98 (73.13)
Employed, *n* (%)	86 (64.18)
Area of residence, *n* (%)	
Urban	104 (77.61)
Current medical therapy, *n* (%)	
5 ASA	104 (77.61)
Corticosteroids	76 (56.72)
Azathioprine	12 (8.95)
Biological therapy	36 (26.9)
Extension, *n* (%)	
L1	16 (11.94)
L2	62 (46.27)
L3	46 (34.33)
L4	10 (7.46)
Behavior	
B1	70 (52.24)
B2	48 (35.82)
B3	16 (11.94)
CDAI, mean ± SD (min–max)	207.07 ± 111.79 (20–526)
CDAI, *n* (%)	
Remission	34 (25.37)
Hb, mean ± SD (min–max)	12.54 ± 2.30 (4.7–17.3)
Anemia, *n* (%)	60 (44.8)
IBDQ total, mean ± SD (min–max)	138.82 ± 31.64 (70–200)
IBDQ1, mean ± SD (min–max)	29.78 ± 7.83 (14–46)
IBDQ2, mean ± SD (min–max)	32.39 ± 8.10 (16–50)
IBDQ3, mean ± SD (min–max)	37.85 ± 9.03 (16–58)
IBDQ4, mean ± SD (min–max)	38.42 ± 7.93 (22–53)

SD: standard deviation; 5 ASA: 5 aminosalycilates; CDAI: Crohn’s disease activity index; IBDQ: Inflammatory Bowel Disease Questionnaire.

**Table 2:** Relationship between the clinical and demographic characteristics of CD patients and anemia.

Characteristic	Anemia	Statistical Measure	Significance *p*
Gender, *n* (%)			
Female	Yes	30 (44.1)	**0.024 ^C^**
	No	38 (55.9)	
Male	Yes	30 (45.5)	
	No	36 (54.5)	
Age, mean ± SD (min–max)			
	Yes	43.37 ± 16.55 (19–82)	0.92 ^t^
	No	43.73 ± 14.96 (23–79)	
Current therapy, *n* (%)			
5 ASA			
Yes	Yes	42 (40.4)	0.18 ^C^
	No	62 (59.6)	
No	Yes	18 (60)	
	No	12 (40)	
Corticosteroids			**0.013 ^C^**
Yes	Yes	44 (57.9)	
	No	32 (42.1)	
No	Yes	16 (27.6)	
	No	42 (72.4)	
Azathioprine			0.11 ^C^
Yes	Yes	8 (66.7)	
	No	4 (33.3)	
No	Yes	52 (42.6)	
	No	70 (57.4)	
Biological therapy			0.40 ^C^
Yes	Yes	14 (38.9)	
	No	22 (61.1)	
No	Yes	46 (46.9)	
	No	52 (53.1)	
Extension, *n* (%)			
L1	Yes	8 (50)	0.75
	No	8 (50)	
L2	Yes	30 (48.4)	
	No	32 (51.6)	
L3	Yes	18 (39.1)	
	No	28 (60.9)	
L4	Yes	4 (40)	
	No	6 (60)	
Behavior, *n* (%)			
B1	Yes	36 (51.4)	0.09
	No	34 (48.6)	
B2	Yes	20 (41.7)	
	No	28 (58.3)	
B3	Yes	4 (25)	
	No	12 (75)	
CDAI, mean ± SD (min–max)	Yes	260.38 ± 116.96 (63–526)	**<0.001 ^M^**
	No	163.85 ± 87.20 (20–358)	
		
CDAI, *n* (%)			
Remission	Yes	4 (11.8)	**<0.001 ^C^**
	No	30 (88.2)	
Activity	Yes	56 (56.0)	
	No	44 (44.0)	

C – Chi square test, t – Student (t) test, M – Mann-Whitney test. The *p*-values below 0.05 are marked in bold.

**Table 3:** Relationship between clinical and demographic characteristics of CD patients and the total score obtained in the IBDQ-32 questionnaire.

Characteristic	No. of Participants	IBDQ–Total Mean (95% Confidence Interval)	*p* Significance
Gender			
Female	68	142.41 (131.75–153.07)	0.33
Male	66	135.12 (123.50–146.75)	
Marital status			
Married	98	139.20 (130.28–148.13)	0.90
Single	36	137.78 (120.84–154.71)	
Area of residence			
Urban	104	137.71 (128.58–146.85)	0.40
Rural	30	142.67 (127.23–158.11)	
Location			
L1	16	147.38 (128.06–166.69)	0.33
L2	62	137.90 (126.09–149.72)	
L3	46	135.78 (121.22–150.35)	
L4	10	144.80 (99.82–189.78)	
Behavior			
B1	70	134.57 (124.28–144.86)	0.09
B2	48	145.50 (131.13–159.87)	
B2	16	137.38 (110.47–164.28)	
CDAI			
Remission	34	152.06 (133.12–170.99)	**<0.001**
Activity	100	134.32 (126.16–142.48)	
Anemia			
Present	60	127.03 (115.70–138.37)	**<0.001**
Absent	74	148.38 (138.47–158.29)	

Note: *p* significances were computed by means of nonparametric Mann–Whitney U test for two sets, respectively, and the Kruskal–Wallis test was used for more than two sets. The *p*-values below 0.05 are marked in bold.

**Table 4:** Influence of anemia on quality of life.

Score	Anemia	Mean ± SD	95% Confidence Interval for Mean		Median	Min	Max	*p* Sig.
			Lower Bound	Upper Bound				
IBDQ_t	No	148.38 ± 29.72	138.47	158.29	153	70	200	**<0.001**
	Yes	127.03 ± 30.36	115.70	138.37	131.50	76	195	
IBDQ_1	No	32.11 ± 7.46	29.62	34.6	32	15	46	**<0.001**
	Yes	26.90 ± 7.42	24.13	29.67	27.50	14	42	
IBDQ_2	No	34.68 ± 7.82	32.07	37.28	34	17	50	**<0.001**
	Yes	29.57 ± 7.66	26.71	32.43	31	16	48	
IBDQ_3	No	40.41 ± 8.65	37.52	43.29	44	16	54	**<0.001**
	Yes	34.70 ± 8.61	31.49	37.91	33.50	22	58	
IBDQ_4	No	40.78 ± 7.49	38.28	43.28	42	22	53	**<0.001**
	Yes	35.50 ± 7.58	32.67	38.33	35	23	49	

Note: *p* significance was computed by means of the nonparametric Mann–Whitney U test. The *p*-values below 0.05 are marked in bold.

**Table 5:** Influence of anemia on quality of life in relation to the activity severity.

CDAI Score/Anemia			*N*	Mean	95% Confidence Interval for Mean		Standard Deviation	Min	Max	*p*
					Lower Bound	Upper Bound				
<150 clinical remission	IBDQ total	Absent	32	157.50	145.59	169.41	33.036	70	200	0.199
		Present	6	129.67	85.34	174.00	42.241	80	174	
	IBDQ-1	Absent	32	34.31	31.34	37.28	8.236	15	46	0.260
		Present	6	29.00	15.76	42.24	12.617	14	42	
	IBDQ-2	Absent	32	36.81	33.75	39.88	8.495	17	50	0.260
		Present	6	31.00	17.37	44.63	12.992	16	45	
	IBDQ-3	Absent	32	43.19	39.88	46.49	9.167	16	54	**0.008**
		Present	6	33.00	23.47	42.53	9.077	22	42	
	IBDQ-4	Absent	32	42.94	39.96	45.92	8.266	22	53	**0.029**
		Present	6	36.00	27.98	44.02	7.642	28	45	
150–220 mild–moderate activity	IBDQ total	Absent	24	147.92	138.46	157.37	22.390	92	189	0.580
		Present	16	141.38	130.21	152.54	20.950	102	161	
	IBDQ-1	Absent	24	31.75	29.10	34.40	6.285	18	46	0.243
		Present	16	29.63	27.03	32.22	4.870	22	38	
	IBDQ-2	Absent	24	34.25	31.52	36.98	6.462	21	46	0.266
		Present	16	32.25	29.86	34.64	4.494	24	40	
	IBDQ-3	Absent	24	40.83	38.03	43.64	6.644	26	48	0.181
		Present	16	39.13	35.60	42.65	6.622	28	45	
	IBDQ-4	Absent	24	40.50	38.03	42.97	5.846	27	48	0.990
		Present	16	40.00	36.74	43.26	6.110	28	46	
221–450 moderate–severe activity	IBDQ total	Absent	18	132.78	120.01	145.54	25.667	91	163	0.268
		Present	30	125.80	114.72	136.88	29.682	89	195	
	IBDQ-1	Absent	18	28.67	25.65	31.68	6.059	17	35	0.185
		Present	30	26.47	23.91	29.03	6.857	16	40	
	IBDQ-2	Absent	18	31.44	27.90	34.99	7.131	21	45	0.285
		Present	30	29.47	26.71	32.23	7.389	19	48	
	IBDQ-3	Absent	18	34.89	31.13	38.64	7.553	24	45	0.990
		Present	30	34.53	31.18	37.89	8.982	24	58	
	IBDQ-4	Absent	18	37.33	33.97	40.69	6.756	27	46	0.305
		Present	30	35.07	32.38	37.76	7.206	25	49	
>450 severe–fulminant activity	IBDQ total	Present	8	101.00	82.03	119.97	22.690	76	131	
	IBDQ-1	Present	8	21.50	16.08	26.92	6.481	14	30	
	IBDQ-2	Present	8	23.50	18.50	28.50	5.976	16	31	
	IBDQ-3	Present	8	27.75	23.64	31.86	4.921	22	34	
	IBDQ-4	Present	8	27.75	23.73	31.77	4.803	23	34	

The *p*-values below 0.05 are marked in bold.

**Table 6:** Effect on quality of life scores by univariate and multiple regressions.

Dependent Variable (Y)	Coefficient of UCDAI Covariate (CI 95%)	Coefficient of Anemia Covariate (CI 95%)	Level of Significance–*p* *t*-Test of Covariates	
			UCDAI	Hb
IBDQ total ^UV^	−0.132 (−0.175 to −0.089)	-	<0.001 *	-
IBDQ total ^UV^	-	−21.345 (−31.579 to −11.111)	-	<0.001 *
IBDQ total ^MR^	−0.112 (−0.159 to −0.064)	−10.561 (−21.110 to −0.011)	<0.001 *	0.05 *
IBDQ1 ^UV^	−0.030 (−0.041 to −0.020)	-	<0.001 *	-
IBDQ1 ^UV^	-	−5.208 (−7.746 to −2.670)	-	<0.001 *
IBDQ1 ^MR^	−0.025 (−0.037 to −0.013)	−2.794 (−5.450 to −0.137)	<0.001 *	0.039 *
IBDQ2 ^UV^	−0.031 (−0.042 to −0.019)	-	<0.001 *	-
IBDQ2 ^UV^	-	−5.109 (−7.751 to −2.467)	-	<0.001 *
IBDQ2 ^MR^	−0.026 (−0.038 to −0.013)	−2.640 (−5.412 to 0.131)	<0.001 *	0.062
IBDQ3 ^UV^	−0.037 (−0.050 to −0.025)	-	<0.001 *	-
IBDQ3 ^UV^	-	−5.705 (−8.649 to −2.76)	-	<0.001 *
IBDQ3 ^MR^	−0.033 (−0.046 to −0.019)	−2.566 (−5.595 to 0.462)	<0.001 *	0.096
IBDQ4 ^UV^	−0.034 (−0.044 to −0.023)	-	<0.001 *	-
IBDQ4 ^UV^	-	−5.284 (−7.853 to −2.715)	-	<0.001 *
IBDQ4 ^MR^	−0.029 (−0.041 to −0.017)	−2.497 (−5.131 to 0.138)	<0.001 *	0.063

Note: *—statistical significance, UV—univariate analysis, and MR—multiple regression.

## Data Availability

The datasets generated during and/or analyzed during the current study are available from the corresponding author on reasonable request.
